# Bidirectional mitochondrial introgression between Korean cobitid fish mediated by hybridogenetic hybrids

**DOI:** 10.1002/ece3.4830

**Published:** 2018-12-21

**Authors:** Ye‐Seul Kwan, Myeong‐Hun Ko, Yeon‐Seon Jeon, Hyo‐Jin Kim, Yong‐Jin Won

**Affiliations:** ^1^ Division of EcoScience Ewha Womans University Seoul South Korea; ^2^ Freshwater Biodiversity Research Bureau Nakdonggang National Institute of Biological Resources Sangju‐si South Korea; ^3^ Department of Life Science Ewha Womans University Seoul South Korea

**Keywords:** hybridogenesis, Korean spined loaches, mitochondrial introgression, unisexual hybridization

## Abstract

Genomic introgression through interspecific hybridization has been observed in some species of the freshwater fish family Cobitidae. Within this family, a* Cobitis hankugensis*–*Iksookimia longicorpa* diploid–triploid hybrid species complex on the Korean peninsula is unique in displaying hybridogenesis, a unisexual reproduction mode that allows hybrids to mediate the transfer of mitochondrial DNA (but not nuclear DNA) between the two parent species. However, populations of the parental species in the wild have never been examined for the potential effect of introgression on their genomes. To address the genetic consequences of unisexual hybridization on the parental species, we examined genetic structure of the two parental species, *C. hankugensis* and *I. longicorpa*, in three independent natural habitats where they coexist with their hybrid complex using DNA sequence data of one mitochondrial gene and three nuclear genes. We found that mitochondrial introgression between the two species was extensive in all the examined localities, while there was no evidence of nuclear introgression across the species boundary. This result indicates that the hybridogenetic individuals mediate mitochondrial introgression from one species to the other, producing mito‐nuclear mosaic genomes such as *C. hankugensis* nuclear genomes associated with *I. longicorpa* mitochondrial DNA and the reverse. The direction and degree of introgression varied among the three localities, but the underlying mechanisms for this observation proved elusive. Introgression might depend on which species serves as the predominant sperm or ovum donor or the environmental conditions of the localities. The present study suggests that introgressive hybridization between pure *C. hankugensis* and *I. longicorpa* species is highly likely where the two species co‐occur with hybridogenetic individuals, but the consequence of introgression could be variable due to the history and environmental characteristics of particular populations across the parental species’ ranges.

## 1 INTRODUCTION

Interspecific hybridization can give rise to unisexual hybrid lineages, which consist of females that clonally inherit their genomes without genetic recombination (Dawley & Bogart, 1989). A variety of unisexual hybrid lineages have been reported in amphibians and reptiles, as well as in fishes, including the families Poeciliidae, Atherinidae, Cyprinidae, and Cobitidae (reviewed in Dowling & Secor, 1997, Vrijenhoek, Dawley, Cole, & Bogart, 1989, Vrijenhoek & Parker, 2009). There are three reproductive models in these unisexual animals: (a) parthenogenesis, where maternal clones are developed without sperm; (b) gynogenesis, where sperm is required to trigger embryogenesis resulting in maternal clones; and (c) hybridogenesis, where fertilization occurs but only the maternal genome is transmitted to the next generation. Depending on the reproductive mode, there may or may not be introgression (gene transfer between parent species via hybrid backcrossing). Parthenogenesis and gynogenesis can effectively prevent genetic introgression between parental species (Choleva et al., [Link]). On the contrary, hybridogenesis can mediate the transfer of mitochondrial DNA (mtDNA) from one species to another without nuclear introgression, thereby producing a mito‐nuclear mosaic genome (Perea, Vukić, Šanda, & Doadrio, 2016; Pløtner et al., 2008; Renoult, Geniez, Bacquet, Benoit, & Crochet, 2009; Saitoh, Kim, & Lee, 2004).

The benthic freshwater fish of the family Cobitidae, commonly called spined loaches, are distributed across Europe, Asia, and part of North Africa (Kottelat, 2012). Species of this family are known to undergo unisexual hybridization in the wild. For example, hybrids with various genetic compositions, including triploids and tetraploids, have been reported in European spined loaches, such as *Cobitis taenia* and its closely related species (Choleva, Apostolou, Rab, & Janko, 2008; Choleva et al., [Link]; Janko et al., 2007). Intensive mating experiments have demonstrated that the hybrids among European spined loaches unisexually reproduce through gynogenesis, in which F_1_ hybrid females generate diploid eggs via aberrant cytological pathways.

**Figure 1:**
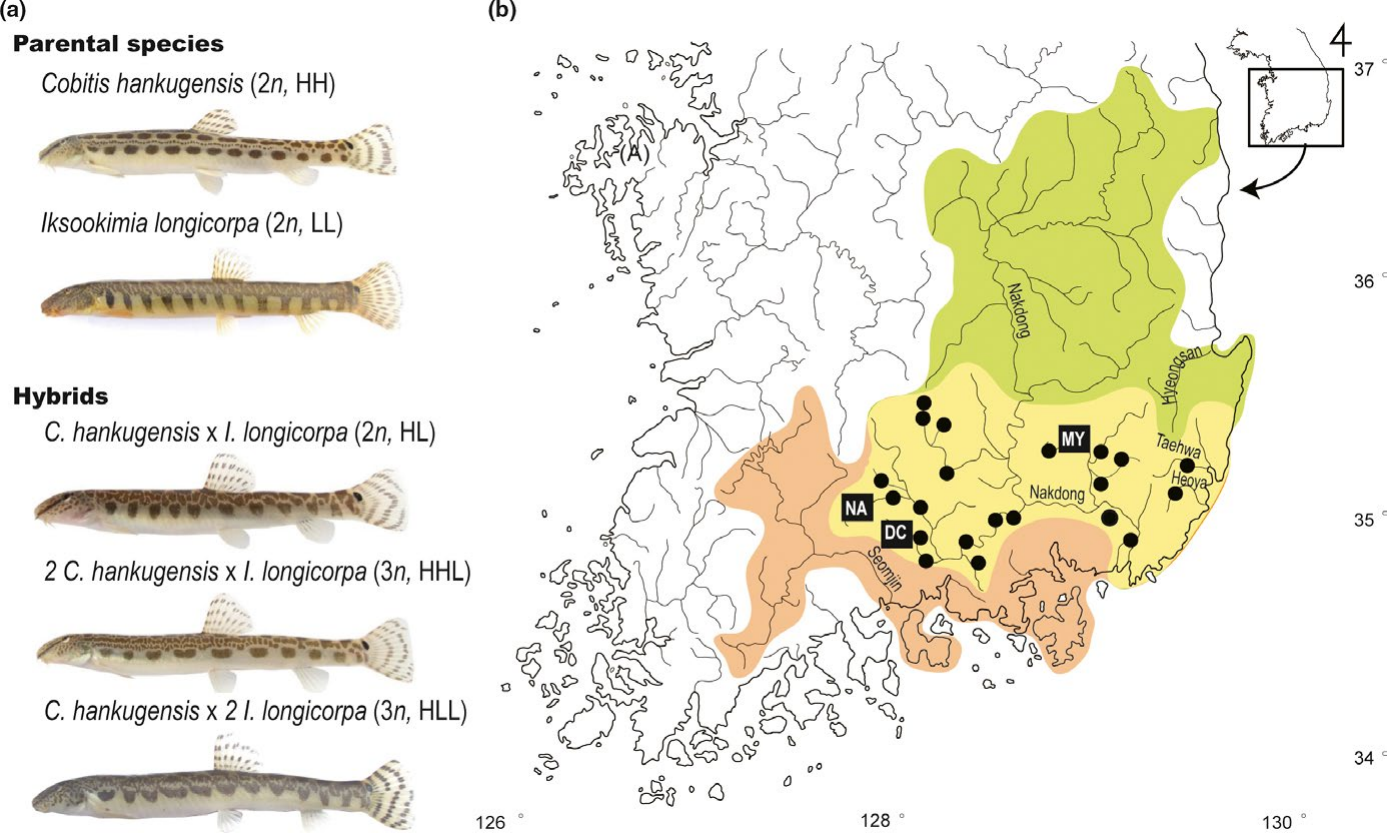
(a) Morphology of *Cobitis hankugensis*, *Iksookimia longicorpa*, and their hybrid complex and (b) their sampling localities in the present study and distribution ranges. Green and orange areas represent the distribution ranges of *C. hankugensis* and *I. longicorpa*, respectively. The yellow area indicates the overlapping distribution range of *C. hankugensis* and *I. longicorpa*. The black dots represent the sites where the hybrid individuals were observed in the previous studies (Ko, 2009; Lee, 1995, 1992). Detailed information on the sampling sites (black squares) and the number of sampled individuals are presented in Table 1

On the other hand, the interspecific hybridization between the two Korean spined loaches, *Cobitis hankugensis* Kim, Park, Son, and Nalbant (2003) and *Iksookimia longicorpa *Kim, Choi, and Nalbant (1976) (Figure 1a), has attracted attention because of its hybridogenetic mode of reproduction, which is unlike that of the European spined loaches. Both of these species are endemic to Korea and are distributed in several rivers and streams flowing southward (Kim, 2009). The ranges of *C. hankugensis* and *I. longicorpa* partially overlap in the Nakdong River (Yellow area in Figure 1). However, since habitat preferences of the two species are different, such as current velocity, types of bottom, and depth of the stream, suitable habitats that the two species tend to coexist are very limited within a river. For this reason, hybrid complexes consisting of a number of females with a few infertile males were found in three localities (squares in Figure 1) with both parental species (Ko, 2009). The hybrids between the two species are classified into three types depending on their karyotype, one diploid (2*n *= 49, referred as HL in Figure 1; H, haploid of *C. hankugensis*; L, haploid of *I. longicorpa*) and two triploids (3*n *= 73, HHL; 3*n *= 74, HLL), while their parental species are diploids (2*n *= 48, *C. hankugensis*; 2*n *= 50, *I. longicorpa*; Kim & Lee, 1995, 1990). Each of the hybrid types and their parental species also showed clear differences in a few morphological characteristics including the lateral banding patterns (Kim & Lee, 1990; Ko, 2009; Figure 1). The proportion of hybrid types were different among localities (Ko, 2009). Previous crossing experiments suggested that this hybrid complex formed through hybridogenesis by demonstrating that the triploid hybrids produce haploid eggs through meiosis after the genome of one parental species is discarded (Kim & Lee, 1995, 2000). For example, triploid hybrids with HHL and HLL type genomes generate haploid eggs of *C. hankugensis* and *I. longicorpa*, respectively (Figure 1). Such a reproduction mode of triploid hybrids has been observed in some kinds of teleost fishes, such as *Squalius alburnoides* (Alves, Gromicho, Collares‐Pereira, Crespo‐Lõpez, & Coelho, 2004) and *Misgurnus* loaches (Morishima et al., 2008).

**Figure 2:**
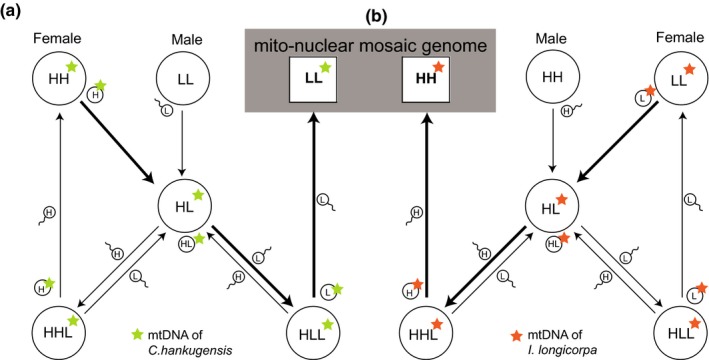
Hypothetical pathways of mitochondrial introgression according to the reproduction mode of the diploid–triploid hybrid complex between *Cobitis hankugensis* and *Iksookimia longicorpa* (Ko, 2009) based on the mother species being either (a) *C. hankugensis* or (b) *I. longicorpa*. Thick lines with an arrow indicate the expected pathway of mitochondrial introgression from one species to the other and squares represent mito‐nuclear mosaic genomes. H: *C. hankugensis* haploid; L: *I. longicorpa* haploid

To date, two hypotheses have been proposed about the reproductive mechanisms between *C. hankugensis* and *I. longicorpa*. First, Kim and Lee (2000) suggested that gene flow does not occur from the triploid hybrids to the parental species. Similarly, hybridogenesis of water frogs (*Pelophylax esculentus*) has been suggested to be a genetic sink for sexual species. However, a recent study showed evidence that the water frog hybrids were able to mediate mtDNA transfer to its parental species (*P. ridibundus*) (Mikulïček, Kautman, Demovič, & Janko, 2014). Interestingly with our studying species, Ko (2009) described how triploid hybrid females could transmit genetic material to the parental species by backcrossing (Figure 2). This second hypothesis was supported by a phylogenetic analysis that showed evidence of mitochondrial introgression: a *C. hankugensis* individual from a hybrid zone was monophyletic with an *I. longicorpa* individual in the same locality (Saitoh et al., 2004).

The present study aims to investigate, using population genetic approaches, the influence of hybridogenesis as a mediator of genetic introgression between *C. hankugensis* and *I. longicorpa* in their natural habitats. For this purpose, three representative localities were selected within which a relatively high abundance of the hybrid complex between *C. hankugensis* and *I. longicorpa* has been maintained with both of their parental species. The genetic structure of the parental species in these three localities was investigated using sequences of one mitochondrial gene and three nuclear genes.

## 2 METHODS

### 2.1 Sampling and DNA extraction

*Cobitis hankugensis* and *Iksookimia longicorpa* individuals were sampled from three independent localities where they coexist with their hybrids (Figure 1). The sampling sites were selected according to previous investigations of the distribution of *C. hankugensis*, *I. longicorpa*, and their hybrids (Ko, 2009; Lee, 1995, 1992). Each sampling site and the number of samples collected are shown in Table 1. Distinctive morphological features of each parental species and their hybrids were used for identification (Kim, 2009): *C. hankugensis* has four clearly visible lines (Gambetta's zone) on the side of the body, and *I. longicorpa* is distinguished by the shape and color of its lateral spots: the first spot is darker than in *C. hankugensis* and the other spots are elongated toward the ventral side (Figure 1). The *C. hankugensis–I. longicorpa *hybrids do not exhibit such morphological features, and they commonly have numerous tiny spots forming an irregular cloud on the dorsal part of the body and a row of oval or rectangular spots running along the lateral sides of the body (Kim & Lee, 1990). According to the previous artificial mating experiment, genome compositions of offspring with different ploidy and chromosome number precisely corresponded to the morphological characteristics (Ko, 2009). Thus, these morphological features allowed us to unambiguously sort out pure parental species, *C. hankugensis* and *I. longicorpa*, from their diploid–triploid hybrids. Only four *I. longicorpa* individuals from the Deokcheon River (DC) were obtained, despite several attempts to collect more samples, due to river maintenance or construction around the site. Genomic DNA was extracted from the pectoral fin of each specimen using Qiagen Tissue and Blood kits (Qiagen, Hilden, Germany) following the manufacturer's protocols.

**Table 1:** Sampling information for the present study. The classification of each species is based on morphological characteristics. Each locality is presented in Figure 1

Abbreviation of study site	Locality (country, province)	Species (*N* a)
NA	Nam River (Namwon, Jeollabukdo) 35°26′N 127°31′E	*Cobitis hankugensis *(11) *Iksookimia longicorpa* (10)
MY	Milyang River (Cheongdo, Gyeongsangbukdo) 36°38′N 128°37′E	*C. hankugensis *(15) *I. longicorpa* (13)
DC	Deokcheon River (Sancheong, Gyeongsangbukdo) 35°16′N 127°50′E	*C. hankugensis *(15) *I. longicorpa* (4)

a Number of individuals examined in this study.

### 2.2 PCR and sequencing

As exchange of mtDNA between *Cobitis* and *Iksookimia* via unisexual reproduction was expected (Saitoh et al., 2004), sequences were obtained from one mitochondrial locus (Cytochrome *b *gene, Cyt *b*) and three nuclear loci (similar to ectodermal‐neural cortex I, ENCI; myosin heavy polypeptide 6, myh6 as described by Li, Ortï, Zhang, and Lu (2007); and Recombination Activating 1, RAG1 as described by Slechtovã, Bohlen, and Tan (2007)). PCR amplification of the Cyt *b* gene and RAG1 gene was performed as previously described by Perdices and Doadrio (2001) and Slechtovã et al. (2007), respectively. The sequences of the other two nuclear loci (ENCI and myh6) were amplified in 20 μl reactions (2 μl of 10X PCR buffer, 1 μl of 2.5 mM dNTPs, 1 μl of 1X bovine serum albumin, 1 μl of each 10 mM primer solution, 0.12 μl (1 unit) of n‐Tenuto *Taq* DNA polymerase (Enzynomics, Korea), 1 μg of genomic DNA, and 12.88 μl of distilled water). The amplified PCR products were used for additional amplifications in 20‐μl PCR reactions that contained 2 μl of 10× PCR buffer, 1 μl of 2.0 mM dNTP, 0.8 μl of each 10 mM nested primer, 0.12 μl (1 unit) of *n*‐Tenuto *Taq* DNA polymerase, 0.5 μl of template and 14.78 μl of distilled water. In the first PCR, each reaction was performed under the following conditions: initial denaturation at 94°C for 2 min, followed by denaturation at 94°C for 40 s, annealing at 53°C for 40 s, and elongation at 72°C for 1 min, which was repeated for 35 cycles with a final elongation step at 72°C for 5 min. These PCR conditions were slightly modified for the inner PCR, in which the annealing temperature was set at 62°C and the number of cycles was 30. All PCR products were sequenced using an ABI 3730xl automatic sequencer (Applied Biosystems, Foster City, CA).

### 2.3 Analysis of DNA sequence data

The obtained sequences were aligned using Clustal W (Larkin et al., 2007), and the accuracy of base determination was confirmed using Geneious version 6.1.4 (Biomatters, Auckland, New Zealand). To solve the phase problem of heterozygotic nucleotide sites in diploid DNA sequences, the sequences of the nuclear genes were processed using the PHASE program (Stephens & Donnelly, 2003; Stephens, Smith, & Donnelly, 2001).

The sequence data were used to determine haplotype using DnaSP v5 (Librado & Rozas, 2009). To confirm the genetic relationships of the haplotypes to *C. hankugensis* and *I. longicorpa*, maximum likelihood (ML) trees were additionally searched using PHYML (Guindon et al., 2010) under the best evolution models, which were selected through the Akaike information criterion (AIC) with MEGA 5.0 (Tamura et al., 2011). Branch support values for each node were estimated by bootstrap analysis with 1,000 pseudoreplicates. Additionally, Cyt *b* sequences were obtained from Genbank for the two parental species (KM576249, *C. hankugensis,* KM576235, *I. longicorpa*), which were collected from distant locations where hybrids between the two parental species have never been observed. These sequences were used as reference sequences in the phylogenetic analyses of the Cyt *b* gene because of the possibility of mitochondrial introgression through hybridization (Choleva et al., 2014; Saitoh et al., 2004). Minimum spanning trees of the haplotypes were constructed using PopART (Bandelt, Forster, & RøHL, 1999; Leigh & Bryant, 2015).

Pairwise *F*_ST_ values among the populations of each species were calculated using the Arlequin program to investigate genetic structure within *C. hankugensis* and *I. longicorpa*. Additionally, analysis of molecular variance (AMOVA) was performed in Arlequin (Excoffier, Laval, & Schneider, 2005) partitioning genetic variation among hierarchical groups. Hierarchical groups were defined in two ways: one was based on locality (NA, DC, MY) and the other was based on species (*C. hankugensis* and *I. longicorpa*). Two different hierarchical schemes were applied to the six samples from the three locations. Parental species were first treated as nested within locality, then locality was treated as nested within parental species.

## 3 RESULTS

**Figure 3:**
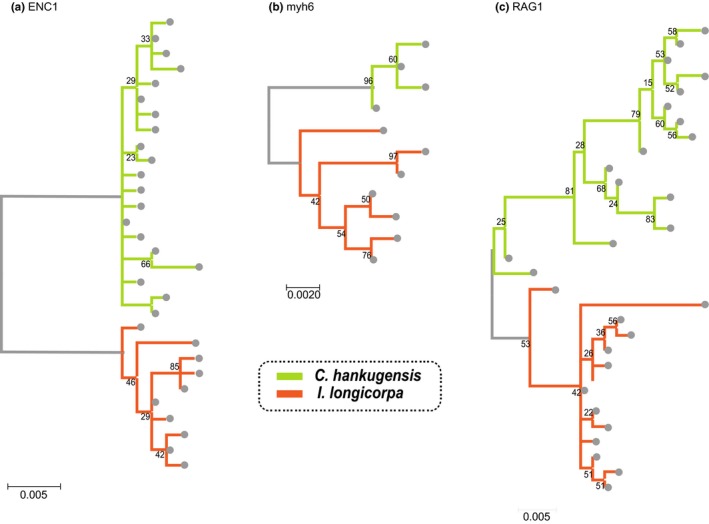
Maximum likelihood trees of haplotypes obtained from the three nuclear genes. CH: *Cobitis hankugensis*; IL: *Iksookimia longicorpa*

**Figure 4:**
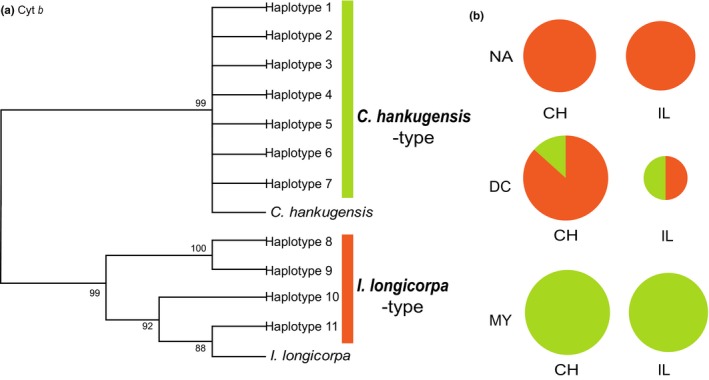
(a) Maximum likelihood tree of Cyt *b* haplotypes including reference sequences obtained from Genbank and (b) haplotype frequencies for the mitochondrial Cyt *b* gene by locality and species. The size of each circle corresponds to the number of examined individuals. CH: *Cobitis hankugensis*; IL: *Iksookimia longicorpa*

We obtained the sequences of the Cyt *b* gene (length 1,027 bp), the ENC1 gene (729 bp), the myh6 gene (677 bp), and the RAG1 gene (781 bp) from each individual (Genbank accessions MH464456–MH464543). All diploid DNA sequences were phased into haplotypes without any ambiguity. The number of unique haplotypes for Cyt *b*, ENC1, myh6, and RAG1 were 11, 30, 11, and 31, respectively. The haplotypes were grouped according to monophyletic relationships in maximum likelihood trees for each gene (Figures 3 and 4). All the nuclear gene trees showed two monophyletic haplotype groups that precisely corresponded to the species from which the haplotype was obtained (Figure 3). However, some of the Cyt *b* haplotypes showed discordance between the species of origin and their genetically assigned type, when we assigned the haplotypes to *C. hankugensis*‐type or *I. longicorpa*‐type based on the genetic closeness to the reference sequences of the two species (Figure 4). For example, *C. hankugensis* individuals with *I. longicorpa* mtDNA were observed in the Nam River (NA) and Deokcheon River (DC). The reverse pattern was also found in the DC River as well as in the Milyang River (MY). In addition, each of the three localities exhibited different haplotype patterns and frequencies of the Cyt *b* gene (Figure 4b). In the Nam River (NA), only *I. longicorpa*‐type haplotypes were observed, while all haplotypes in the Milyang River (MY) were *C. hankugensis*‐type, regardless of the morphological differences between individuals. The population from the Deokcheon River (DC) was composed of both types of haplotypes.

Pairwise *F*_ST_ was calculated to estimate genetic differentiation among populations, which were defined by species and locality (Table 2). All the resulting *F*_ST_ values among six populations based on the two nuclear loci, ENC1 (Table 2A) and RAG1 (Table 2C), were statistically significant. In the nuclear myh6 gene, *F*_ST_ values were significant among *I. longicorpa* populations, but not significant among *C. hankugensis* populations (Table 2B), because the haplotypes from *C. hankugensis* were separated from a single dominant haplotype by only one or two mutational steps (Supporting Information Figure S1). Genetic differentiation estimated using the mitochondrial Cyt *b* was also generally significant among populations but was not significant between *C. hankugensis* and *I. longicorpa* populations within any of the investigated localities (NA, *F*_ST_ = 0.1200; DC, *F*_ST_ = −0.1258; MY, *F*_ST_ = 0.1100; Table 2D). To test whether such genetic differentiation among populations is correlated with species identity (*C. hankugensis* and *I. longicorpa*) or geographic locality (NA, DC, and MY), analysis of molecular variance (AMOVA) was conducted. However, all the resulting Φ_CT_ values among the groups (locality and species) were nonsignificant, regardless of the loci (Table 3). On the contrary, the genetic differentiation among populations within groups was supported by statistical tests, except for when the testing groups were defined by locality with the mitochondrial Cyt *b* locus (Φ_SC_ = −0.0362, *p* = 0.57; Table 3).

**Table 2:** Population pairwise *F*‐statistics. The probability of the test statistic *p* < 0.0042 after Bonferroni correction

(A) Cyt *b*						
	CH_NA	IL_NA	CH_DC	IL_DC	CH_MY	IL_MY
CH_NA						
IL_NA	0.1200					
CH_DC	0.7623*	0.6962*				
IL_DC	0.6889*	0.5736*	−0.1258			
CH_MY	0.8327*	0.7670*	0.8095*	0.7840*		
IL_MY	0.4855*	0.4116*	0.5153*	0.3314*	0.1100	

(B) ENC1						
	CH_NA	IL_NA	CH_DC	IL_DC	CH_MY	IL_MY
CH_NA						
IL_NA	0.8006*					
CH_DC	0.3658*	0.3081*				
IL_DC	0.7390*	0.3638*	0.0950*			
CH_MY	0.4743*	0.4649*	0.1423*	0.2796*		
IL_MY	0.7321*	0.5303*	0.2673*	0.3538*	0.4213*	

(C) myh6						
	CH_NA	IL_NA	CH_DC	IL_DC	CH_MY	IL_MY
CH_NA						
IL_NA	0.8658*					
CH_DC	0.1454	1.0000*				
IL_DC	0.6022*	0.3123*	0.8501*			
CH_MY	0.0419	0.9218*	0.0172	0.7343*		
IL_MY	0.6795*	0.8027*	0.8349*	0.5410*	0.7621*	

(D) RAG1						
	CH_NA	IL_NA	CH_DC	IL_DC	CH_MY	IL_MY
CH_NA						
IL_NA	0.6421*					
CH_DC	0.1520*	0.5703*				
IL_DC	0.2097*	0.7099*	0.1842*			
CH_MY	0.2864*	0.6047*	0.2283*	0.2158*		
IL_MY	0.2541*	0.5947*	0.2313*	0.1757*	0.2578*	

Note

NA, DC, and MY indicate investigated localities (see Table 1). CH: *Cobitis hankugensis*; IL: *Iksookimia longicorpa*.

* Statistical significance at α = 0.05.

**Table 3:** Φ‐statistics resulting from AMOVA tests grouped by locality (NA, DC, MY) and species (*Cobitis hankugensis* and *Iksookimia longicorpa*)

Gene	ENC1		myh6		RAG1		Cyt *b*	
Group	Locality	Species	Locality	Species	Locality	Species	Locality	Species
Among groups (Φ_CT_)	−0.8623 (*p* = 0.87)	0.9017 (*p* = 0.09)	−0.7006 (*p* = 0.67)	0.7745 (*p* = 0.10)	−0.6442 (*p* = 0.09)	0.7659 (*p* = 0.10)	0.8704 (*p* = 0.06)	−0.4500 (*p* = 1.00)
Among populations within groups (Φ_SC_)	0.9516*	0.5063*	0.9379*	0.7194*	0.8720*	0.4525*	−0.0362 (*p* = 0.57)	0.8679*
Among six populations (Φ_ST_)	0.9099*	0.9515*	0.8944*	0.9367*	0.7896*	0.8718*	0.8657*	0.8085*

Note

Φ_SC_ for locality groups represents genetic differentiation between the two parental species within the same locality. On the other hand, Φ_SC_ for species groups represents genetic differentiation among the three of geologically distant localities within the same species.

* Statistical significance at α = 0.05.

## 4 DISCUSSION

Interspecific hybridization between *C. hankugensis* and *I. longicorpa* has been recognized in several tributaries of the Nakdong River and adjacent rivers on the Korean peninsula. Previous artificial mating experiments suggested that the hybrid complex of the two parental species, including diploid or triploid hybrid females, reproduce by hybridogenesis. Although the hybridogenetic mode of reproduction can theoretically induce genomic introgression from one parental species to another via their hybrids (Choleva et al., 2014; Saitoh et al., 2004), the genetic consequences of unisexual hybridization between the two parental species, *C. hankugensis* and *I. longicorpa*, have not been examined in natural populations. In the present study, population genetic structure was investigated in *C. hankugensis* and *I. longicorpa* from three natural habitats (Figure 1 and Table 1) where the two species co‐occur with their hybrids.

Results showed that all the haplotypes of the nuclear loci were clearly distinguished by the species from which they were obtained (Figure 3). In contrast, all of the investigated localities showed either complete or partial inconsistency between the species‐types assigned by mitochondrial Cyt *b* haplotypes and the morphological species identification (Figure 4). Surprisingly, the Cyt *b* locus exhibited insignificant genetic differentiation between *C. hankugensis* and *I. longicorpa* populations within each of the hybrid localities (Tables 2 and 3). Genetic discordance between mitochondrial and nuclear loci can be attributed to genetic introgression through hybridization (Toews & Brelsford, 2012; Wallis et al., 2017). Therefore this observation indicates the existence of mitochondrial introgression between the two parental species. It is unlikely that the mitochondrial genes of two divergent species retain ancestral polymorphism while their nuclear genes exhibit reciprocal monophyly (Avise, 2009; Avise et al., 1987). We therefore rule out the hypothesis that the difference of genetic pattern in Cyt *b* gene results from incomplete lineage sorting. Moreover, some of the same mitochondrial haplotypes were observed in both of the parental populations within the investigated localities (Supporting Information Table S1). Therefore, these results support the hypothesis of mitochondrial introgression between the two parental species without nuclear gene introgression through backcrossing from the hybridogenetic hybrids to one of the parental species as proposed by Ko (2009), Figure 2. The hybridogenetic mode of reproduction may explain the reason why the hybrids are distributed only in the very limited area in which the two parental species coexist (Figure 1b). If the hybrids reproduce by parthenogenesis or gynogenesis, the hybrids will endure relatively long periods when there is no or a single parental species present. However, the hybridogenetic hybrids between *C. hankugensis* and *I. longicorpa* cannot produce offspring without the males of the parental species because male hybrids are infertile. According to the model of hybridogenesis presented in Figure 2, even if the hybrids reproduce with only one of the parental species, the ultimate genome compositions of the hybrids (HL, HHL, and HLL) will be diluted out being gradually replaced with either HH or LL depending on which species is the sperm donor. Therefore, in order for a hybrid complex population to be stably sustained, the hybrids must coexist with both parental species. This constraint may explain the ephemeral nature of the diploid–triploid hybrid species complex in time as well as the patchiness of its contemporary distribution in the Nakdong River like islands. Consequently, it can be presumed that such a hybrid complexes have intermittently formed in places where two parental species came into contact in the past and have subsequently gone extinct due to the cessation of contact. Previous studies (Ko, 2009; Lee, 1995, 1992) reported that hybrid individuals were observed in some localities where only one parental species co‐occurred (black dots in Figure 1b), which seems to indicate not only the birth of diploid–triploid hybrid complexes in the past but also ongoing processes of the potential extinction of them due to the absence of the other parental species.

With respect to the two possible directions of mitochondrial introgression between the two species as illustrated in Figure 2, population data of Cyt *b* showed that the direction and strength of the introgression varied by geographic locality. Both the NA and MY localities showed unidirectional introgression of the mitochondrial genome from one species to the other, but the direction of introgression between the species was different among the two population. According to the haplotype patterns (Figure 4), the mtDNAs of *I. longicorpa* infiltrated into *C. hankugensis* in the NA locality, while the mitochondrial introgression occurred from *C. hankugensis* to *I. longicorpa* in the MY locality. On the other hand, the DC locality yielded two representative types of mitochondrial haplotypes in both parental species (Figure 4b) despite the small number of *I. longicorpa* individuals sampled (*N* = 4), supporting bidirectional introgression. These different patterns of introgression in the natural hybrid populations raise the question about what factors govern the direction and degree of the introgression between *C. hankugensis* and *I. longicorpa*.

A variety of prezygotic and postzygotic factors can influence the frequency and direction of hybridization (Wirtz, 1999). In the case of hybridization between Atlantic salmon (*Salmo salar*) and brown trout (*S. trutta*), all of the hybrids in southern European rivers were the product of crosses between female salmon and male trout, due to a fitness disparity in nuclear‐mtDNA incompatibility with respect to development time (Álvarez, Garcia‐Vazquez, & Taylor, 2011). In contrast, hybrids between male salmon and female trout have been observed in Canadian rivers, possibly due to an abundance of sexually mature Atlantic salmon (McGowan & Davidson, 1992). As the hybridogenetic hybrids between *C. hankugensis* and *I. longicorpa* serve as a mediator between the two parental species (Figure 2), the direction of the introgression may be determined by which species predominantly acts as a mother or father species in two independent events: producing the hybrids and backcrossing to the parental species. To assess the biological factors governing the directionality and degree of introgression between *C. hankugensis* and *I. longicorpa*, artificial mating experiments were once conducted testing the outcome of all the possible combination of mating pairs. The result of these experiments showed that all of the pairs were capable of successful fertilization and development (Ko, 2009), indicating no detectable difference in fitness among progeny with different genomic backgrounds under laboratory conditions. This experimental result is consistent with the present observations in that the introgression of mitochondria across the species boundary can occur in any direction between the species even in wild populations.

Alternatively, we might find a clue for the question from the population history of both parental species and their hybrids in nature. Though studies on the ecology and demography of hybrid populations of *C. hankugensis* and *I. longicorpa* are scarce, one study was conducted in the NA locality (Ko, 2009), where it was observed that mitochondrial introgression occurred unidirectionally from *I. longicorpa* to *C. hankugensis*. According to Ko (2009), the frequency of male *C. hankugensis* was almost three times higher than that of male *I. longicorpa*, except for few of infertile hybrid males. The female hybrids were more frequently observed than the females of the other species (*C. hankugensis *[HH]:*I. longicorpa *[LL]:hybrids [HL, HHL, and HLL] = 1:1:2). Therefore, the mating rate between female hybrids and male *C. hankugensis* is expected to be higher than other possible mating pairs, leading to the backcrossing to one of the parental species, *C. hankugensis *(Figure 2b). Importantly, the number of HHL hybrids was the highest among three types of hybrids in the NA locality, and HHL hybrids were more morphologically similar to *C. hankugensis* (Figure 1). Environmental conditions of the NA locality, including the presence of a sandy bottom, are more suitable for *C. hankugensis* than *I. longicorpa *(Ko, 2009). Under these environmental conditions, individuals of the HHL type may have higher fitness than other type of hybrids in the NA locality, although the fitness difference between the morphological characteristics explained by natural habitat conditions is not known. Interestingly, the MY locality where mitochondrial introgression occurred unidirectionally from *C. hankugensis* to *I. longicorpa *is characterized by a rocky riverbed which *I. longicorpa* generally prefers. The riverbed of the DC locality where bidirectional mitochondrial introgression occurred contains both sands and rocks approximately in equal portions. These environmental differences in the three localities suggest that hybrid female mating choice between males of the parental species might explain the morphological characteristics that result from backcrossing and lead to asymetrical introgression (Peters, Myers, Dudaniec, O'Connor, & Kleindorfer, 2017; Stein & Uy, 2006). Therefore, we expect that the direction of backcrossing and thus its resultant direction of mitochondrial introgression could be linked to the abundance of male species and/or a female choice, depending on environmental conditions.

Acknowledging the effect of backcrossing on the direction of mitochondrial introgression, we can next consider the role of female species in producing diploid hybrids as another factor for directional mitochondrial introgression. When backcrossing occurs into *C. hankugensis* as in the NA locality, if the *I. longicorpa* predominantly acted as a mother species (Figure 2b), the mtDNA of *C. hankugensis* would be replaced more rapidly with that of *I. longicorpa* as observed in the present study (Figure 4b). On the other hand, if more *C. hankugensis* females significantly contributed to producing diploid hybrids (Figure 2a), *I. longicorpa* with *C. hankugensis*‐type mtDNA would gradually accumulate, unless there were competing contributions of *I. longicorpa* females. Therefore, the unidirectional mitochondrial introgression from *C. hankugensis* to *I. longicorpa* in the MY locality might have been affected by the composition of the parental species, similar to that of the NA locality, but leading to a different outcome (Figure 2a). However, the result of DC locality reveals a kind of balance among competing factors acting on the alternative pathways of mitochondrial introgression, suggesting that the mitochondrial introgression between *C. hankugensis* and *I. longicorpa* does not always end up with complete replacement of one species’ mitochondria with that of the hybridizing partner species under certain conditions.

In conclusion, the findings of this study indicate that mitochondrial introgression through the unique reproductive mode of hybridogenesis may have occurred between *C. hankugensis* and *I. longicorpa* in extensive areas wherever the two species coexist with their hybrid complexes. Although the present study reveals the direction and degree of introgression can be varied among the localities with hybrid complexes, the identification of primary factors affecting the direction of introgression requires further study. Currently, little is known about the reproductive ecology and behavior, the demographic histories of *C. hankugensis*, *I. longicorpa*, and the persistence of their diploid–triploid hybrids in natural habitats. To develop a better understanding of the process of hybridization and introgression, which varies with geographical regions, future work should be conducted throughout the full distributional range of the parental species and hybrid complexes.

## CONFLICT OF INTEREST

The authors declare that they have no competing interests.

## AUTHOR CONTRIBUTIONS

YSK and YJW designed the experiment. MHK, YSK, YSJ, and HJK collected and identified samples. YSK, YSJ, and HJK generated and analyzed the genetic data. YSK and YJW led the writing of the manuscript. All authors contributed critically to the drafts and gave final approval for publication.

## Data Availability

All DNA sequences of this study were deposited at Genbank (MH464456–MH464543).
