# Supplementary material for: Bidirectional mitochondrial introgression between Korean cobitid fish mediated by hybridogenetic hybrids
Source: Ecol Evol. 2018 Dec 21;9(3):1244–54. doi: 10.1002/ece3.4830 (PMC6374646; doi:10.1002/ece3.4830)

**Supplementary figure 1.** Minimum spanning networks for three nuclear genes: (A) ENC1 (B) RAG1 and (C) myh6 genes. White and black circles represent haplotypes from *C. hankugensis* and *I. longicorpa*, respectively. A small bar between the circles indicates one mutation. The size of each circle corresponds to the relative haplotype frequency.


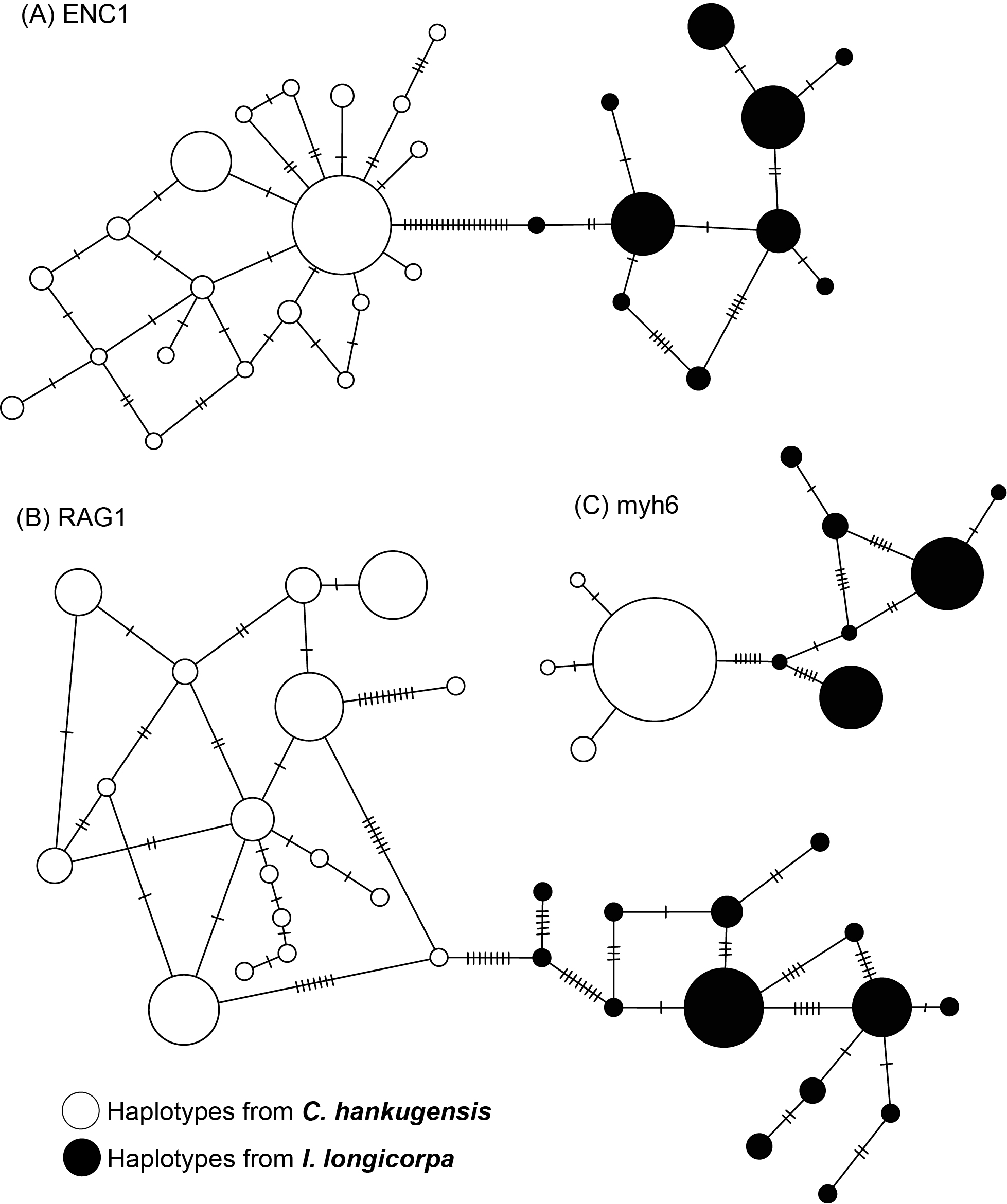

Supplement: Supplementary file 1 [file ECE3-9-1244-s001.docx]
