# Supplementary material for: Bidirectional mitochondrial introgression between Korean cobitid fish mediated by hybridogenetic hybrids
Source: Ecol Evol. 2018 Dec 21;9(3):1244–54. doi: 10.1002/ece3.4830 (PMC6374646; doi:10.1002/ece3.4830)
Supplement: Supplementary file 2 [file ECE3-9-1244-s002.docx]

**Supplementary table 1.** Haplotype frequencies of each parental species, *C. hankugensis* (CH) and *I. longicorpa* (IL), within each of the localities. The haplotypes were corresponded with Figure 4A.

| Locality | NA | | DC | | MY | |
| --- | --- | --- | --- | --- | --- | --- |
| Species | CH | IL | CH | IL | CH | IL |
| Haplotype 1 |  |  |  |  | 14 | 7 |
| Haplotype 2 |  |  | 2 | 2 |  |  |
| Haplotype 3 |  |  |  |  | 1 | 2 |
| Haplotype 4 |  |  |  |  |  | 1 |
| Haplotype 5 |  |  |  |  |  | 1 |
| Haplotype 6 |  |  |  |  |  | 1 |
| Haplotype 7 |  |  |  |  |  | 1 |
| Haplotype 8 | 9 | 8 |  |  |  |  |
| Haplotype 9 | 2 |  |  |  |  |  |
| Haplotype 10 |  | 2 |  |  |  |  |
| Haplotype 11 |  |  | 13 | 2 |  |  |
